# Dual-energy CT for gastrointestinal bleeding

**DOI:** 10.1259/bjro.20220054

**Published:** 2023-03-22

**Authors:** Miyuki Okamura-Kawasaki, Yuya Uesugi, Satoshi Yabusaki

**Affiliations:** 1 Department of Radiology, Tomakomai City Hospital, Shimizu-cho, Tomakomai-shi, Hokkaido, Japan; 2 Department of Radiological Technology, Tomakomai City Hospital, Shimizu-cho, Tomakomai-shi, Hokkaido, Japan

## Abstract

Dual-energy computed tomography (DECT) can be used for various types of analyses, including iodine quantification, and its usefulness in diagnosing gastrointestinal diseases has been reported. This pictorial review describes the use of DECT in the diagnosis of gastrointestinal bleeding.

Virtual non-contrast computed tomography (CT) is available in DECT and can be used as a substitute for pre-contrast CT in the case of gastrointestinal haemorrhage. The omission of pre-contrast CT can reduce radiation exposure by approximately 30%. A low-keV virtual monochromatic X-ray image (VMI) can increase the contrast of iodine, and iodine maps can provide better visibility of extravasation. These analytical images can provide a diagnosis with a high degree of confidence. In addition, the low-keV VMI clearly illustrates the vascular structure, which may be useful for improving the visibility of vascular lesions and for confirming the arterial anatomy before embolisation. Considering that these analytical images are created on the basis of contrast-enhanced CT, the positional information of organs is entirely identical, thus allowing the comparison of images regardless of intestinal peristalsis or body motion.

In conclusion, the analytical images of DECT can solve the problems of conventional protocols, and DECT is considered useful in the imaging diagnosis of gastrointestinal bleeding.

## Introduction

Gastrointestinal bleeding is a relatively common and may sometimes have a severe outcome. Endoscopy is usually the first choice for diagnosis; contrast-enhanced computed tomography (CT) is often performed before endoscopy because of its simplicity and ease of use without pre-procedure.^1,2^ Given that the symptoms of upper and lower gastrointestinal bleeding often overlap, CT may be the first choice even when it is difficult to identify the responsible site of bleeding from the symptoms.

Dual-energy CT (DECT), which has become popular in the past decade, is capable of various analyses (*e.g.,* iodine quantification), and its usefulness for diagnosing gastrointestinal diseases has been reported.^2,3^ Although DECT can reduce radiation exposure and provide high-contrast images, it is essential to understand the appropriate analysis method in advance because DECT analysis is an active process. This article will outline the value of DECT in the diagnosis of gastrointestinal bleeding.

## Standard CT protocol in the diagnosis of gastrointestinal bleeding

**Figure 1.**
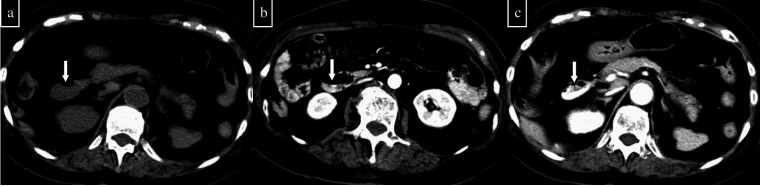
An 87-year-old woman had progressive anaemia and underwent pre-contrast and post-contrast two-phase (arterial and delayed phase) CT. (a) On the pre-contrast CT, it is difficult to determine whether the duodenal contents (arrow) are haematomas. (b) The arterial phase shows extravasation into the duodenal lumen. (c) Extravasation increases in the delayed phase. On the basis of these findings, duodenal active bleeding is suspected.

Gastrointestinal bleeding is classified into upper and lower bleeding, and these two types of bleeding are separated by the ligament of Treitz. The symptoms of gastrointestinal bleeding include haematemesis, bloody stools, and occult blood. Generally, the standard CT protocol for suspected gastrointestinal bleeding cases is multiphase imaging, pre-contrast CT, and post-contrast two-phase CT (Figure 1).^1,4,5^

Pre-contrast CT is adequate for identifying intestinal haematomas; uncoagulated blood generally Exhibits 1–16 HU, whereas coagulated blood typically Exhibits 16–41 HU.^4^ These two types of blood are often difficult to differentiate from other high-density intestinal contents such as food residues, drugs, or surgical instruments. After a contrast agent is applied, a diagnosis is made by imaging in two phases, namely, the arterial phase and the delayed phase, to identify the extravasation of the contrast agent and its spread. The arterial phase allows the confirmation of extravasation and is useful for determining the responsible vessel. The delayed phase confirms the increase or spread of extravasation into the intestinal tract.

Several problems exist with the conventional standard protocol. The diagnosis of gastrointestinal bleeding may be difficult when the amount of bleeding is minor. The dislocation of organs in each phase caused by respiration or bowel movements exists, and the interpretation of multiphase images may be difficult for inexperienced clinicians. Increased radiation exposure is also a problem, but multiphase imaging unavoidably increases the radiation dose.

## Analysis of DECT

**Figure 2.**
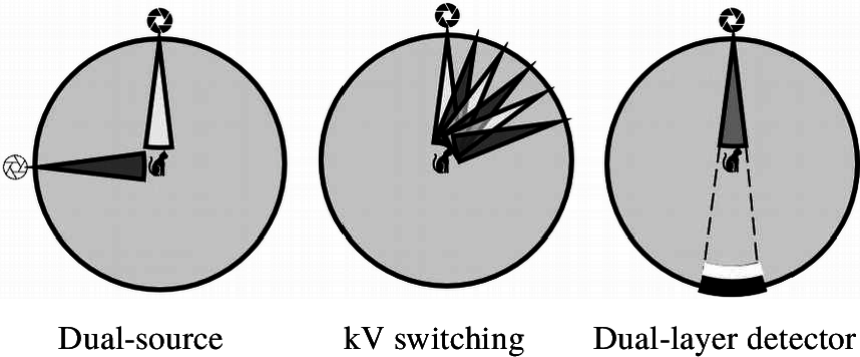
Various methods of DECT. DECT with dual-source, kV switching, and dual-layer detector are simulated from left to right. DECT, dual-energy computed tomography.

DECT uses two types of X-rays with different tube voltages or a dual-layer detector to perform CT imaging (Figure 2), which can differentiate between several substances, such as iodine or calcium, and produce a variety of analysed images. Different vendors use different image acquisition methods; however, there is no significant difference in the analysis that can be performed. This article focuses on the virtual non-contrast CT (VNC) image, virtual monochromatic image (VMI), and iodine map, which are especially useful in diagnosing gastrointestinal bleeding. All images were obtained by a 64-slice dual-layer detector DECT (IQon spectral CT, Philips, Amsterdam, the Netherlands). The standard protocol of contrast enhanced CT using 530 mg/kgI of contrast agent was implemented. The injection time for single-phase contrast enhanced CT was 50 s, and the scan started 20 s after the end of the injection. The injection time for two-phase contrast enhanced CT was 30 s, and the arterial phase was performed 10 s after the end of injection. Delayed phase was initiated 80 s after the end of injection.

## Omission of pre-contrast CT with VNC images

**Figure 3.**
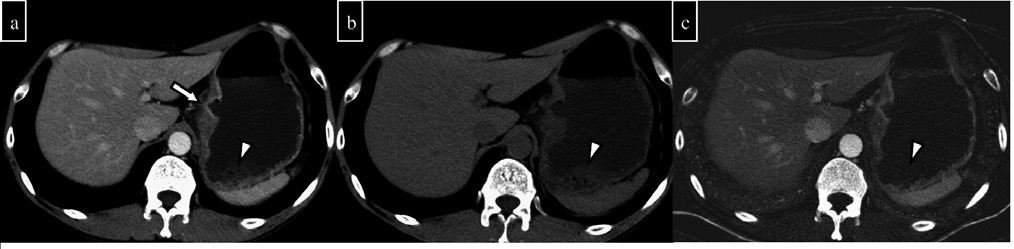
A 52-year-old man has a history of gastric ulcer bleeding. Owing to haematogenous vomiting, single-phase CT was performed. (a) Conventional CT shows an ulcer in the gastric lesser curvature (arrow). Gastric contents show slightly high density on conventional CT (arrowheads). (b) VNC shows a slightly high density of gastric contents (arrowheads). (c) The iodine map shows no apparent contrast agent content in the gastric contents. Haematoma is suspected rather than active bleeding. No obvious bleeding was observed on the endoscopy. VNC, virtual non-contrast CT.

**Figure 4.**
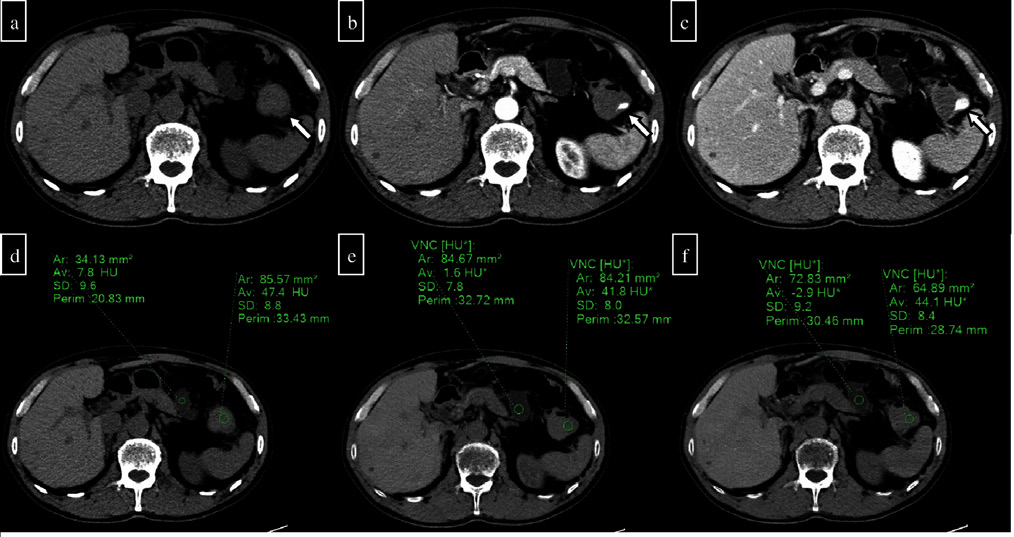
A 70-year-old man presented to the clinic for bleeding after the endoscopic mucosal resection of a colonic polyp and underwent pre- and post-contrast two-phase CT (arterial and delayed phases). (a) Pre-contrast CT showed a relatively high density of contents in the splenic flexure of the colon, thus suggesting a haematoma (arrow). (b) The arterial phase shows extravasation in the splenic flexure. (c) Extravasation is increasing in the delayed phase. From the above, active haemorrhage after endoscopic mucosal resection was suspected. (d) Pre-contrast CT measurement shows a 47.4 HU in the splenic flexure lumen and a 7.8 HU in the small intestine lumen in the area nearby. (e) In the VNC created from arterial phase images, the densities of the splenic flexure and the small intestine in the nearby area are 41.8 and 1.6 HU, respectively. (f) In the VNC created from the delayed phase image, the densities in the splenic flexure and the small intestine in the nearby area are 44.1 and -2.9 HU, respectively. There is no significant difference that would quantitatively affect the evaluation. VNC, virtual non-contrast CT.

A VNC image is an image reconstructed by subtracting the iodine content from contrast-enhanced image data. Given that a VNC image can virtually create a pre-contrast CT, it is possible to evaluate the presence or absence of iodine content in areas showing high density even when a pre-contrast CT has not been performed (Figure 3). In addition, considering that a VNC image is an image created from post-contrast CT, its positional information of organs is completely identical to that of post-contrast CT compared with pre-contrast CT, thus allowing the comparison of images without being affected by respiration or bowel movements. The contrast is not entirely identical to that of pre-contrast CT; however, there is no significant difference in diagnostic performance between pre-contrast CT and VNC in gastrointestinal bleeding (Figure 4 ).^6,7^ Therefore, it is possible to omit pre-contrast CT in cases where gastrointestinal bleeding is suspected. Compared with the imaging protocols of pre-contrast CT and post-contrast two-phase, the protocol of post-contrast two-phase and VNC construction reduces radiation exposure by approximately 30%, and a significant reduction in radiation exposure is expected with the use of VNC.^6,7^

## Improved visualisation of gastrointestinal bleeding with VMI and iodine map

**Figure 5.**
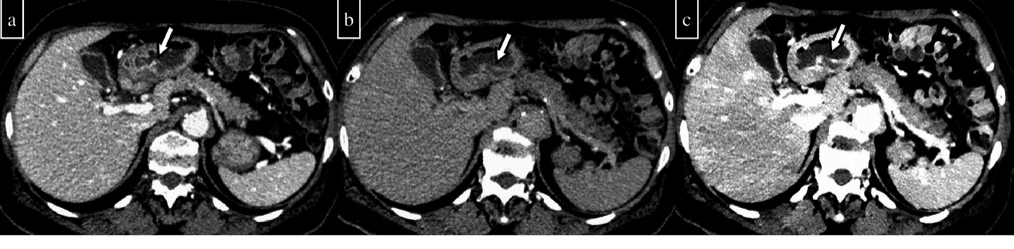
A 62-year-old woman presented with complaints of black stools and hypotension to the hospital. A contrast-enhanced two-phase (portal venous phase and delayed phase six minutes later) CT was performed to assess the cause of bleeding. (a) In the portal venous phase, a punctate high-density area was found near the gastric pylorus (arrow). (b) A punctate high-density area is seen near the gastric pylorus in the delayed phase, and the high-density area is slightly enlarged compared with that in the portal venous phase. This is considered an active haemorrhage. (c) The VMI (40 keV) of the delayed phase shows a clear spread of contrast agent compared with the original image. VMI, virtual monochromatic image.

A VMI is a virtual image obtained at an energy of approximately 40–200 keV. Low-keV images have increased contrast enhancement because of the increased attenuation of the contrast agent.^8^ Even in patients with poor renal function, in whom contrast agent dosage is reduced, or in cases of unexpected contrast failure, it is occasionally possible to obtain images that are similar to those of conventional contrast-enhanced CT. In the case of gastrointestinal bleeding, the use of a VMI allows for more definite extravasation, thus improving the identification of the bleeding area (Figure 5). By contrast, low-keV images increase image noise and metal artefacts.^2,3^

**Figure 6.**
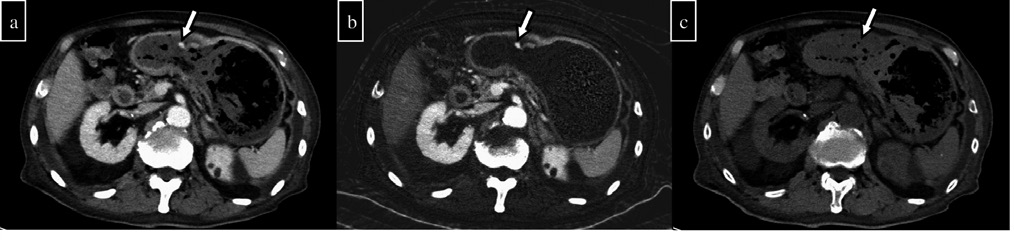
An 81-year-old man was hospitalised for heart failure because of atrial fibrillation. A single-phase contrast-enhanced CT was performed because of sudden haematochezia. (a) Conventional CT shows a high-density area in the stomach (arrow). (b) The iodine map shows high density in the same area and low density in all other gastric contents. (c) VNC shows a decrease in the density of the high-density area in the stomach on conventional CT. From (b) and (c), the presence of active bleeding in the stomach can be diagnosed. VNC, virtual non-contrast CT.

An iodine map is an image in which pixel numbers are expressed in mg/mL iodine density instead of CT values, and areas containing a contrast agent are depicted as high intensity. The quantitative evaluation allows for the easy determination of the contrast between intestinal contents and extravasation (Figure 6).^6,8^

VMIs and iodine maps improve viability by more clearly depicting extravasation, and similar to VNC images, the location information of the organs is identical, thus allowing for a more confident diagnosis. These images increase reliability and diagnostic performance, especially when determining the absence of gastrointestinal bleeding.^5^

## VMI for confirming arterial anatomy

**Figure 7.**
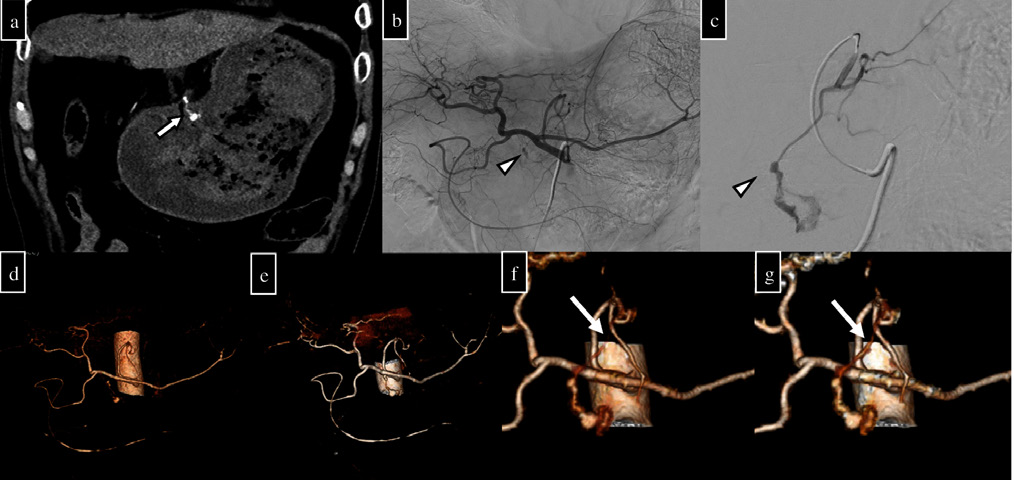
A 63-year-old man. Eight days after omental plugging for gastric perforation, he developed progressive anaemia and hypotension. Emergency endoscopy was performed, and a large amount of blood was found in the stomach. Given that endoscopic haemostasis was difficult, embolisation was decided, and pre- and post-contrast CT (arterial and delayed phases) were performed to identify the bleeding spot. (a) In the arterial phase (coronal section), an ulcer on the gastric kyphosis and a hyperintense area near the ulcer were observed, and extravasation was considered (arrow). (b) Abdominal arteriography and (c) left gastric arteriography show extravasation from the peripheral left gastric artery branch (arrowhead). (d) and (e) Vascular VR images created by conventional CT of the arterial phase and VMI (40 keV) images of the arterial phase with bone removal; no responsible vessels are seen in either case. (f) and (g) VR images created after extraction of the left gastric artery vessels using conventional CT of the arterial phase and VMI (40 keV) images of the arterial phase; in both images, the responsible branch is indicated (dashed arrows). The branches are more clearly depicted in the VR images created using VMI (40 keV) in both methods. VR, volume rendering; VMI, virtual monochromatic image.

Low keV images increase the density of the contrast agent, thus allowing for the vascular structures to be more clearly visualised. Therefore, in cases of contrast reduction or those wherein arterial phase images are not available and arterial delineation is not clear, it is possible to clarify the vessel outlines. This may be useful for improving the visibility of vascular lesions that cause gastrointestinal bleeding and for confirming arterial anatomy before embolisation (Figure 7).^2,3^

## Conclusion

This article reviews the basics of diagnostic imaging in gastrointestinal bleeding and the functional analyses of DECT, which can reduce radiation exposure when creating VNC images and omitting non-contrast CT, improve the visibility of extravasation when creating iodine maps and VMIs, and provide a more precise depiction of vascular anatomy using VMIs. If CT imaging with DECT is available, DECT would be a great advantage if it is the imaging modality of choice for gastrointestinal bleeding. DECT may become the gold standard for diagnosing gastrointestinal bleeding in the future.
